# The Practice of Medicine

**Published:** 1905-01

**Authors:** 


### THE PRACTICE OF MEDICINE.

Judge Davis of the Court of Quarter Sessions of Philadelphia, in his charge to the jury in the case of one Thomas E. Eldridge, says:

"The practice of medicine consists in the offering of service and assuming the responsibilities of treating diseases, deformities and injuries, no matter by what means this is professed to be done.

"Every State, because of the inherent nature of the calling of medicine, possesses the right both constitutionally and legally to demand a standard degree of qualification which will protect citizens from the consequences of incompetency and unskilled practice.

"Anyone practicing medicine without the license of the State, which license is on the part of the State a guarantee of the possession of the qualification to safely pursue medical practice, is an illegal practitioner.

"Everyone, therefore, who offers service as outlined, no matter by what means he professes to treat diseases, deformities and injuries, must, as a condition precedent thereto, have obtained the license in accordance with the laws of the commonwealth."

The practice of medicine consists of the offering of service as a physician and the assumption of the responsibility of treating disease. The means used is not a matter of vital importance and the attempted evasion of the law by the defense that the alleged doctor did not prescribe drugs, etc., was seen to be a fallacy by the impartial judge. The trial developed the fact that the defendant had a diploma from an institution called the Eastern College of Electro-Therapeutics, and that he was practicing on the basis of that authority, pretending to be exempt from the medical practice law of the State of Pennsylvania. This calls attention again to some institutions which, although they pretend to give regular courses of instruction in certain branches, are little better than diploma mills, in that they turn out half-educated men who are prejudiced against the science of medicine, in its broad sense, and who are ready to evade the laws of the State wherever they require more education than these would-be doctors possess.

The courts of several States have also decided as above, notably Missouri, Alabama, and Illinois.

---

Every member of the State Medical Association who reads this issue of the "Red Back" is urged to bring to the attention of his Senator and Representative the article "A Brief for the People," published as leading editorial, and see that he reads it. It will be profitable to discuss it with the members of the Legislature, for although it is a pretty full presentation of the subject there is much that could not be gone into in so brief a space. For instance, a moment's reflection will show that the clause in the Practice Act which exempts from examination and license "those who do not give drugs or medicines," not only entrusts, without the slightest restrictions, the dangerous privilege of practicing medicine to those who claim that they have medical diplomas and to be educated in the fundamental branches of medicine, but it is a premium offered to any and all to engage in practice, without any qualification whatever,—a bid to all the quacks driven out of other States to come to Texas. Under this clause your hostler, your shoemaker,—the day laborer,—the most utterly ignorant man or woman, white, black or yellow—has *carte blanche* to practice medicine in all its branches without let or hindrance, and the State will protect him in collecting his bill—the only possible restriction is that he shall not "give drugs or medicines." The State has no means of knowing—does not ask, nor seem to care to know,—whether the man thus entrusted with human lives can even read and write. It is infamous; a disgrace to the State and an outrage on the people. It must be repealed.

